# PYK2 is overexpressed in chronic lymphocytic leukaemia: A potential new therapeutic target

**DOI:** 10.1111/jcmm.17688

**Published:** 2023-02-06

**Authors:** Francesca Vittoria Sbrana, Benedetta Fiordi, Jessica Bordini, Daniela Belloni, Federica Barbaglio, Luca Russo, Lydia Scarfò, Paolo Ghia, Cristina Scielzo

**Affiliations:** ^1^ Malignant B cells biology and 3D modelling Unit, Division of Experimental Oncology IRCCS Ospedale San Raffaele Milan Italy; ^2^ School of Medicine Università Vita‐Salute San Raffaele Milan Italy; ^3^ B‐cell neoplasia Unit, Division of Experimental Oncology IRCCS Ospedale San Raffaele Milan Italy

**Keywords:** chronic lymphocytic leukaemia, focal adhesions, microenvironment, targeted therapy

## Abstract

Chronic Lymphocytic Leukaemia (CLL) is the most common adult B‐cell leukaemia and despite improvement in patients' outcome, following the use of targeted therapies, it remains incurable. CLL supportive microenvironment plays a key role in both CLL progression and drug resistance through signals that can be sensed by the main components of the focal adhesion complex, such as FAK and PYK2 kinases. Dysregulations of both kinases have been observed in several metastatic cancers, but their role in haematological malignancies is still poorly defined. We characterized FAK and PYK2 expression and observed that PYK2 expression is higher in leukaemic B cells and its overexpression significantly correlates with their malignant transformation. When targeting both FAK and PYK2 with the specific inhibitor defactinib, we observed a dose–response effect on CLL cells viability and survival. In vivo treatment of a CLL mouse model showed a decrease of the leukaemic clone in all the lymphoid organs along with a significant reduction of macrophages and of the spleen weight and size. Our results first define a possible prognostic value for PYK2 in CLL, and show that both FAK and PYK2 might become putative targets for both CLL and its microenvironment (e.g. macrophages), thus paving the way to an innovative therapeutic strategy.

## 1 INTRODUCTION

Chronic lymphocytic leukaemia (CLL) is the most common form of leukaemia in the western world within adults. It is a lymphoproliferative disorder involving progressive accumulation of mature CD19^+^ CD5^+^ B lymphocytes in the Peripheral Blood (PB), Bone Marrow (BM) and other lymphoid organs such as the Spleen (SP) and Lymph Nodes (LN).^1^ The clinical features of CLL show high heterogeneity among patients; this variability could be explained by both intrinsic factors (e.g. genetics and epigenetics) and external stimuli. Despite the introduction of new effective targeted therapies such as BTK (e.g. ibrutinib, acalabrutinib) and BCL‐2 (e.g. venetoclax) inhibitors, a majority of patients eventually relapse, so CLL still remains an incurable disease.^2^, ^3^

A major role in the development of the disease and the onset of drug resistance is played by the crosstalk between the malignant clone and the Tumour Microenvironment (TME). Malignant B cells recirculate between lymphoid organs and the bloodstream but while cells in the PB become quiescent, B lymphocytes in tissues find a supporting niche and actively proliferate.^4^ In the specific case of CLL, main actors and mechanisms through which the TME regulates the leukaemic clone progression still need to be fully elucidated.^5^

We and others previously demonstrated that the trafficking and homing of malignant B cells is tightly regulated by cytoskeletal reorganization^6^ and by different cells in the TME that contribute to CLL survival and progression.^7^ In particular, the survival of CLL cells is supported by the direct contact with nurse‐like cells (CLL‐specific tumour associated macrophages^5^) and targeting macrophages lead to indirect CLL cells apoptosis and inhibition of disease progression.^8^ Along this line, recent evidence shows that there is a complex interaction between cancer cells and the TME through an exchange of mechanical perturbations sensed by surface ‘sensor’ receptors, which translate these inputs into chemical signals. The Focal Adhesion complex is one of these sensors, and its main components are the Focal Adhesion Kinase (FAK) and its related homologue protein Proline‐rich Tyrosine Kinase 2 (PYK2) also known as PTK2 and PTK2B, respectively. These are non‐receptor protein kinases, which play an important role in several cellular processes, beside mechanosensing and cell adhesion or migration, including proliferation and apoptosis. While FAK is expressed in almost all tissues, PYK2 is expressed mainly in haematopoietic cells.^9^ Both FAK and PYK2 dysregulations have been observed in different metastatic cancers, but their role and expression in haematological malignancies and specifically in leukaemia, is not well defined yet.^10^, ^11^ In a genome‐wide study, PYK2 has been found to correlate with improved outcomes in CLL patients under chemoimmunotherapy regimen.^12^ More recently FAK has been proposed as a modulator of migration and invasion in CLL.^13^

In a pre‐clinical setting, it has been previously demonstrated that targeting FAK and/or PYK2 could bring some benefits in some haematological cancers such as Multiple Myeloma,^14^ Myelodysplastic Syndromes,^15^ Acute Myeloid Leukaemia,^16^ BCR/ABL‐transformed model of Chronic Myeloid Leukaemia^17^ and in Mantle Cell Lymphoma (MCL). In particular, it has been demonstrated that MCL cells, upon culture with stromal cells, show an activation of pathways such as NF‐kB, Akt, c‐Myc, Cyclin‐D and p42/44, all associated with cancer progression, and FAK inhibition abrogates the activation of all these effectors. The same happens after the occurrence of resistance to the BTK inhibitor ibrutinib, providing a rationale for the administration of specific FAK inhibitors in combination with ibrutinib in MCL.^18^

Inhibitors exist and target focal adhesion tyrosine kinases on ATP binding sites inhibiting their catalytic activity to repress the auto‐phosphorylation activity in Tyr397 on FAK and Tyr402 on PYK2. A second‐generation inhibitor VS‐6063 (i.e. defactinib or PF‐04554878) by Verastem Oncology, with the same mechanism of action, has already been proven safe and effective in patients in several clinical trials for solid tumours.^19^, ^20^, ^21^, ^22^ As of October 2022, 7 clinical trials are currently ongoing for defactinib testing, for a total of more than 20 studies overall, but only one is investigating defactinib effects on haematological malignancies.^23^

In this work, we aim at defining FAK and PYK2 expression in CLL cells and exploring the effect of defactinib administration to CLL, both in vitro (2D and 3D culture) and in vivo, to understand how leukaemic cells could exploit mechano‐transduction pathways like FAs recruitment via FAK and PYK2 activation, to sustain their development, progression and resistance to therapy.

## 2 MATERIALS AND METHODS

### 2.1 Cell cultures and human primary sample purification

The HS5 human bone marrow stromal cell line was obtained from the American Type Culture Collection (ATTC) and cultured in DMEM medium (GIBCO) supplemented with 10% v/v FBS, 15 mg/mL Pen/Strep at 37°C and 5% CO2. Primary leukaemic, from patients, and healthy, from donors, CD19^+^ B cells were negatively selected from fresh PB and BM using the RosetteSep B lymphocyte enrichment kit (Stemcell Technologies) and separated via density gradient Lymphoprep (Stemcell Technologies). Healthy B cells were then isolated with EasySep Cell isolation kit (Stemcell Technologies). B cells were isolated from lymphoid tissues after mechanical smashing to recover the cells in suspension and later purified following the same protocol described for healthy B cells. The purity of all the isolated B cells was >98%. Primary cells were cultured in RPMI 1640 medium (EuroClone) supplemented with 10% (v/v) Fetal Bovine Serum (FBS) and 15 mg/mL Pen/Strep (complete RPMI) at 37°C and 5% CO2.

### 2.2 RNA extraction, reverse transcription and Real Time PCR (RT‐qPCR)

RNA extraction was performed according to the manufacturer's instructions, using ReliaPrep RNA Cell Miniprep System (Promega). In general, the RNA is collected after consecutive centrifugation steps and isopropanol/DNAse solution washes and centrifugation steps, then resuspended in a variable amount of nuclease‐free water. cDNA was synthesized according to the manufacturer's protocol using the RevertAid H Minus First Strand DNA Synthesis kit (Thermo Fisher Scientific). RT‐qPCR analysis was performed using Titan HotTaq Probe qPCR mix (BioAtlas) in an ABI7900 Thermal Cycler instrument (Applied Biosystem). The analysis was performed in duplicate or triplicate. Quantification of PTK2 (FAK) and PTK2b (PYK2) probes transcripts (Applied Biosystem) was performed according to the Ct method, using YWHAZ as the housekeeping gene.^24^

### 2.3 Protein lysis and Western Blot (WB)

Cells were lysed on ice for 15 min in RIPA Buffer (Sigma‐Aldrich) with fresh protease and phosphatase inhibitors cocktail (Roche). Cells were then centrifuged at 13,200 rpm for 15 min at 4°C, and supernatants were collected and stored at −80°C until further use. Protein content was determined using Micro BCA protein assay kit (Thermo Scientific), according to the manufacturer's instructions. The total of protein content of about 10 × 10^6^ cells was supplemented with NuPage Sample Buffer (4×) and NuPage Sample Reducing Agent (10×) and loaded onto 4–12% sodium dodecyl sulphate‐polyacrylamide gradient gels (Invitrogen), then transferred to nitrocellulose membranes (Thermo Scientific). Membranes were blocked for 1 hour in PBS‐Tween containing 5% BSA and incubated overnight with primary antibodies, followed by species‐specific Horseradish Peroxidase (HRP)‐conjugated secondary antibodies (diluted 1:5000) for 1 h. Membranes were probed with the following primary antibodies: Akt (Cell Signalling, Rabbit, 1:1000), pAkt S473 (Cell Signalling, Rabbit, 1:1000), p27 (Santa Cruz, Mouse, 1:500), Fak (Cell Signalling, Rabbit, 1:1000), pFak Y397 (Cell Signalling, Rabbit, 1:1000), PYK2 (Cell Signalling, Mouse, 1:1000) and pPYK2 Y402 (Cell Signalling, Rabbit, 1:1000). All WB were normalized to anti β‐Actin HRP conjugated (Cell Signalling, Rabbit, 1:50,000). Amersham ECL Western Blotting Analysis System from GE Healthcare was used to visualize immuno‐reactive bands. Western blots were acquired using Bio‐Rad Chemidoc (Bio‐Rad) and quantification of relative protein expression levels was performed using Fiji ImageJ dedicated tool (https://imagej.net/software/fiji/). Graphs and statistical analysis were performed using GraphPad Prism (https://www.graphpad.com/scientific‐software/prism/).

### 2.4 Immunofluorescence (IF) microscopy

Glass slides were coated with Poly‐ornithine for 20 min at 37°C. They were then washed with H20 Milli‐Q and PBS. CLL primary cells were seeded with a concentration of 1 × 10^6^ cells/slide and let adhere for 2 h. PFA4% was used to fix cells for 15 min and PBS washes were then made. Cells were permeabilized and blocked with a solution of 10% FBS, 0,3% Triton x‐100 (Sigma‐Aldrich) and 1 mg/mL of BSA. Fluorescence staining were done by leaving primary antibodies o/n at 4°C, secondary antibodies 2 h at room temperature and DAPI (1:2000) for 5 min. Then slides were mounted with ProLong Gold Antifade Reagent (Invitrogen) and let dry for 24 h. Images were acquired on Olympus FluoVIEW 3000 RS Confocal microscope (Olympus) with a 30× (NA 1.05) silicone objective. Primary antibodies used: PYK2 (Cell Signalling, 1:200), pPYK2 (Y402) (Cell Signalling, 1:200), FAK (Cell Signalling, 1:200) and pFAK (Y397) (Thermo Fisher, 1:200). Secondary antibodies: Alexa 488 (1:500). Ten images for each condition and patients were acquired and analysed with Fiji ImageJ. A macro was created to measure the mean grey value of the fluorescence signal of the different markers in order to obtain a quantification of the levels of expression of the protein of interest. Graphs and statistical analysis were performed using GraphPad Prism (https://www.graphpad.com/scientific‐software/prism/).

### 2.5 3D scaffolds preparation, co‐culture and treatments in RCCS™ bioreactor

For CLL 3D co‐culturing conditions, the protocol previously adapted to CLL in our lab has been followed.^25^ Briefly: Gelatine disks of 4 mm of diameter and 3 mm height were cut from Spongostan™ gelatine sheets (Ethicon) using a sterile biopsy punch and then pre‐seeded with BM derived stromal cells HS5 (200.000/scaffold) for 4 h in 96‐well suspension culture plate (Greiner Bio‐one). Scaffolds were then transferred to 10 mL High Aspect Ratio Vessels (HARV) in 600 μL DMEM culture medium supplemented with 10% v/v FBS and cultured overnight in the RCCS™ bioreactor. The day after, CLL cells were added to the vessels, following the optimal CLL cells:stromal cells ratio, set in previous experiments.^25^ After 5 h, vessels were filled with growth medium (RPMI 1640 culture medium supplemented with 20%). After 48 h of 3D dynamic culture in the bioreactor, supernatants were withdrawn from the vessels and centrifuged at 1500 rpm for 5 min. Recovered cells were counted. Clarified supernatants were put again in the vessels with or 4 μM defactinib (Verastem Oncology). Cultures were stopped after 4 h of treatment and cells in the supernatants and in the scaffolds were recovered and submitted to Trypan Blue exclusion test for viability and cell count.

### 2.6 Flow cytometry analysis

For cell culture flow cytometry analysis cells were recovered, washed with 3 mL of either PBS (Euroclone) or binding buffer (PBS + 5%FBS) and centrifuged at 1500 rpm for 5 min. We next added the following antibodies: Annexin V‐FITC (Thermo Fisher, 5 μL/tube), PI (Thermo Fisher, 10 μL/tube). We incubated for 20 minutes at room temperature in the dark. We then washed with 3 mL of PBS or binding buffer and resuspended the sample in 500 μL PBS. Samples were read at the flow cytometer (Navios; Beckman Coulter). Data were analysed by FCS express software. For in vivo mice experiments. Cells from PB, SPs, BM, lymph nodes and peritoneal washes from each mouse were divided into three tubes, they were washed with 3 mL of PBS (Euroclone) and centrifuged at 1500 rpm for 5 min. Staining continued as follows: CD19‐PC7 (BioLegend, 1 μL), CD5‐APC (BioLegend, 1 μL), CD11b‐PE‐Cyanine7 (BD, 1 μL). Samples were read at the flow cytometer (Navios; Beckman Coulter). Data were analysed by FCS express software.

### 2.7 Cell titre viability assay

The assay was performed according to the manufacturer kit (Cell Titre Glo Luminescent Cell Viability Assay, Promega). CLL cells were plated 3 × 10^6^/mL in their culture medium within a 96‐opaque‐walled plate in a volume of 100 μL. Control wells were prepared adding only medium. Then, an equal amount of Cell Titre Glo Reagent was added to each well and the two parts were mixed for 2 min on a shaker. The multiwell was then left 10 min at room temperature to stabilize the signal. Finally, luminescence was recorded with a Luminometer. The results were compared to an ATP standard curve previously generated.

### 2.8 2D co‐cultures and cells treatments

Pre‐coating with either 200 × 10^3^ HS5 stromal cells/well was done by seeding cells the day before the start of the co‐cultures. 100 × 10^3^ CLL/mL were added in suspension onto plates, stromal cells, endothelial cells, with 4 μM defactinib (Verastem Oncology) for 48 h. At the indicated time‐points, CLL cells were recovered and analysed by flow cytometry for viability.

### 2.9 Mouse model and treatments

Eight‐week‐old C57BL/6 female mice (Charles River Laboratories) were injected intraperitoneally with 10^7^ cells purified from SP of a 12‐month‐old Eμ‐TCL1 leukaemic mouse (on a C57BL/6 background) by the EasySep B‐cell enrichment kit (StemCell Technologies). The purity of transplanted cells, checked by flow cytometry, was higher than 95%. Mice were monitored weekly for leukaemia development by flow cytometric analysis of the PB samples. Treatment was initiated 6 weeks after transplant (day 0) when a range of 10%–40% of CD19^+^CD5^+^ cells was detected in the PB of transplanted mice. Mice were divided in two groups: control and treated with defactinib. Defactinib (Verastem Oncology) was dissolved in a vehicle solution of 0.5 g Carboxy‐Methyl Cellulose, 100 μL tween‐80 and filled up to 100 mL with dd‐H2O. The compound was administered at a concentration of 75 mg/kg by oral gavage on a twice‐a‐day schedule, every day for 3 weeks. The control group received the vehicle with the same schedule. At the time of sacrifice, PB, SPs, BM, lymph nodes and peritoneal washes cells were isolated and analysed by flow cytometry. While liver, lungs and kidneys were fixed and stored in paraformaldehyde 4% for future immunohistochemistry analysis. Full counts of white blood cells in each lymphoid compartment were counted with Trypan Blue exclusion test for viability. Spleens were checked in weight and dimension. Complete Blood Count (CBC) was also performed to assess the blood compartment. Flow cytometry analysis was performed as previously mentioned.

### 2.10 Statistical analysis

One‐way anova, Two‐way anova and paired or unpaired Student's *T* tests were performed for statistical analysis (GraphPad Prism v.9.0a) (*p = <0.05; **p = <0.01; ***p = <0.001; ****p = <0.0001).

### 2.11 Human and mouse ethics statements

Patients with CLL were diagnosed according to the updated National Cancer Institute Working Group (NCIWG) guidelines.^26^ PB samples were obtained after informed consent from patients who were untreated or off treatment for at least 6 months. The study was approved by the Ospedale San Raffaele (OSR) ethics committee under the protocol VIVI‐CLL entitled: ‘In vivo and in vitro characterization on CLL’. Clinical and biological characteristics of patients with CLL who provided samples for the experiments are reported in Table 1. The buffy coats study was approved by the Ospedale San Raffaele (OSR) ethics committee under the protocol Leu‐Buffy_coat entitled: ‘Characterization of leukocyte subpopulations from buffy coats’. Eμ‐TCL1 transgenic mice were provided by J. C. Byrd (Ohio State University, Columbus, OH) on a C3H/B6 background and were backcrossed for >10 generations to C57BL/6 N background.^27^ 8 weeks old wild‐type (WT) C57BL/6 N mice were supplied by Charles River Laboratories. Mice were maintained in a specific pathogen‐free animal facility and treated in accordance with European Union and approval of the Ospedale san Raffaele Institutional Ethical Committee.

**TABLE 1:** Table summarizing the clinical and biological parameter for all the patients used in the experiments (na = not available)

Patient no	Surface CD38 result	Progression result	IGHV identity
1	2.3	Stable	100
2	na	Progressive	100
3	5	Stable	100
4	15.90	Stable	98.30
5	91.64	Stable	100
6	17.8	Stable	99.7
7	5.3	Progressive	100
8	29.2	Stable	100
9	na	Stable	100
10	na	Progressive	100
11	15.8	Progressive	100
12	0.1	Stable	88.4
13	na	Stable	93.7
14	4.6	Stable	89.7
15	0.57	Stable	92.36
16	0.30	Stable	97.28
17	0.30	Stable	93.60
18	na	Stable	96.50
19	0.17	Stable	92.71
20	na	Progressive	91.30
21	0.2	Stable	94.49
22	1.00	Progressive	93.15
23	0.50	Stable	95.79
24	0.07	Stable	88.42
25	3.2	Stable	97.90
26	19.10	Stable	na
27	na	na	99.70
28	5	Stable	na
29	87.7	Progressive	100.00
30	5	Stable	100
31	0.3	Stable	97.28
32	5.9	Progressive	96.22
33	0.6	Stable	86
34	0.12	Stable	93.75
35	0.1	Stable	na
36	0.1	Stable	100
37	5	Stable	100

## 3 RESULTS

### 3.1 FAK and PYK2 are differentially expressed in healthy B and CLL cells

**FIGURE 1:**
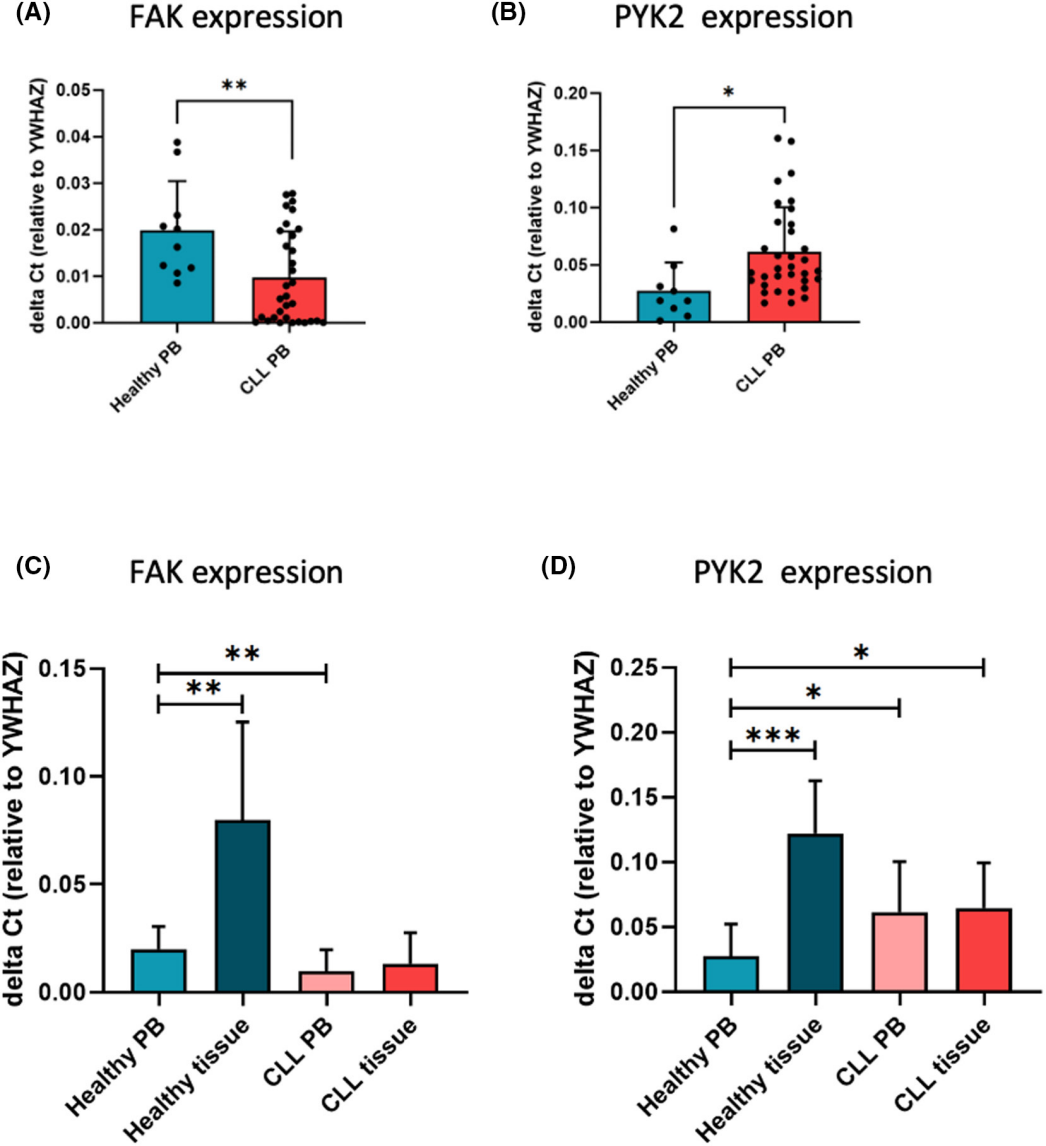
FAK and PYK2 expressions. (A and B) FAK and PYK2 mRNA expression analysis in primary B cells from healthy (*n* = 10) and CLL (*n* = 36) samples determined by RT‐qPCR and compared to YWHAZ expression. FAK was found more expressed in healthy samples compared to CLL ones (*p* = 0.0079), while PYK2 was found more expressed in CLL samples compared to healthy ones (*p* = 0.0175). (C and D) FAK and PYK2 mRNA expression analysis in PB‐derived and tissue‐derived B cells isolated either from healthy donors (*n* = 10 PB, *n* = 3 tissue) or patients with CLL (*n* = 36 PB, *n* = 14 tissue). Both FAK and PYK2 mRNA levels are significantly higher in B cells isolated from healthy tissues compared to those derived from the PB (FAK *p* = 0.0015; PYK2 *p* = 0.0006). This difference is not observed for CLL B cells (FAK *p* = 0.3524; PYK2 *p* = 0.8066)

We investigated FAK and PYK2 mRNA expression levels in B cells obtained from the peripheral blood (PB) of patients with CLL (*n* = 36, Table 1) or healthy donors (*n* = 10; Figure 1A,B). While FAK showed significantly higher expression level in PB‐derived healthy B‐cell samples (*p* = 0.0079) compared to CLL (Figure 1A), PYK2 showed the opposite trend, with significantly higher expression levels in CLL PB‐derived cases (*p* = 0.0175) compared to healthy B cells (Figure 1B). We analysed and compared FAK and PYK2 mRNA expression in lymphoid tissues derived from both healthy individuals and CLL cases where possibly their expression could have a major impact in the interaction with the tissue microenvironment. For this analysis, 3 healthy lymphoid tissues (tonsils *n* = 3) and 14 tissue samples isolated from patients with CLL (BMs *n* = 7; SPs *n* = 4; LNs *n* = 3) were used. Both FAK and PYK2 showed significantly higher mRNA levels of expression in B cells isolated from healthy tissues compared to those derived from the healthy PB (FAK *p* = 0.0015; PYK2 *p* = 0.0006; Figure 1C,D). This difference has not been observed for CLL B cells where the level of expression remains the same in the different compartments (FAK *p* = 0.3524; PYK2 *p* = 0.8066 Figure 1C,D). These results suggest that the expression of both kinases is finely regulated in different anatomical compartments in healthy conditions while this regulation appears to be lost in CLL as suggested for other cancers.^28^

### 3.2 FAK and PYK2 expression correlates with the clinical outcome

**FIGURE 2:**
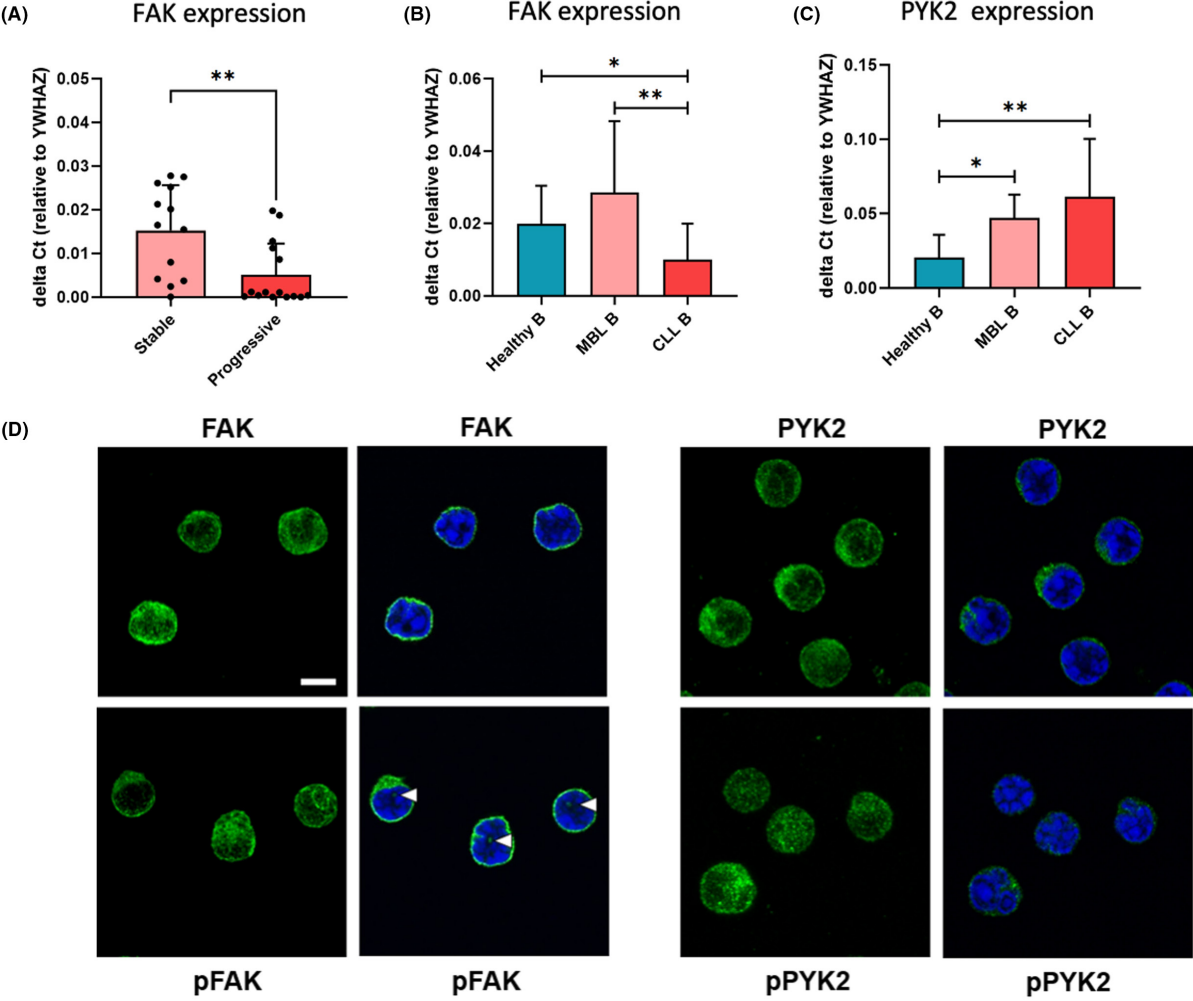
FAK and PYK2 expression correlates with the clinical outcome. (A) FAK mRNA expression analysis in samples derived from the PB of patients diagnosed as stable compared to the progressive ones: FAK expression is significantly (*p* = 0.0051) lower in the progressive samples. (B and C) FAK and PYK2 mRNA expression analysis in healthy B (*n* = 10), MBL (*n* = 5), CLL (*n* = 30) samples. PYK2 mRNA expression was determined by RT‐qPCR and compared to YWHAZ expression. FAK expression does not show a significance correlation with B cells progression towards malignancy, while PYK2 expression significantly correlates with CLL development form a healthy status (healthy B) (healthy B vs. MBL *p* = 0.0116; healthy B vs. CLL *p* = 0,0063). (D) Representative confocal images of CLL cells stained for nucleus (Hoechst, Blue) and for FAK, pFAK, PYK2 or pPYK2 (Alexa 488, green). Morphology of FAK and PYK2 is represented as a Z stack. A single slide was merged with Hoechst signal (blue) to point out the presence of the signal in the nuclei (white arrows). Scale bar: 5 μm

We observed a high variability of expression for both kinases among patients with CLL, thus we analysed the results in relation to the clinical and biological characteristics of the patients (Table 1). We observed that lower FAK expression significantly correlated with a progressive disease for patients with CLL (Figure 2A; *p* = 0.0051), while we could not find any correlations with the clinical and biological parameters for PYK2 in our cohort of patients. To further understand FAK and PYK2 role in CLL pathogenesis, we decided to study their expression at different stages of the disease natural history. We compared FAK and PYK2 mRNA levels in B lymphocytes collected from the PB of healthy donors (*n* = 10), patients with the pre‐leukaemic state MBL (*n* = 5)^29^ and patients with overt CLL (*n* = 36; Figure 2B,C).^30^ For FAK, we observed that in MBL cases its expression is close to healthy B cells (Figure 2B), while for PYK2 we showed that in MBL cases its expression is significantly higher with respect to healthy B cells (healthy B vs. MBL *p* = 0.0116, Figure 2C). These results suggest that a dysregulated balance of expression of the two kinases can occur during disease progression. We further investigated FAK and PYK2 localization and expression in CLL cells by immunofluorescence analysis (4 patients, 10 images each, Figure 2D). As expected, we found both kinases expressed mainly in the cytoplasm of CLL cells. In parallel, we observed a constitutive activation of both kinases, in particular pFAK (Y397) was observed also in the nuclei of some CLL cells while p‐PYK2 (Y402) was more punctuated in podosome‐like structures^31^ (Figure 2D) possibly suggesting a different role or regulation for the two proteins.

### 3.3 Defactinib treatment of CLL cells induces apoptosis in vitro and downregulation of survival associated pathways

Based on our observations of PYK2 overexpression and activation in CLL cells, we aimed at testing whether the pharmacological inhibition of PYK2, might have any relevant therapeutic effect. To this aim, we exploited the potent and selective ATP‐competitive FAK/PYK2 inhibitor defactinib.^19^, ^20^, ^21^, ^22^

**FIGURE 3:**
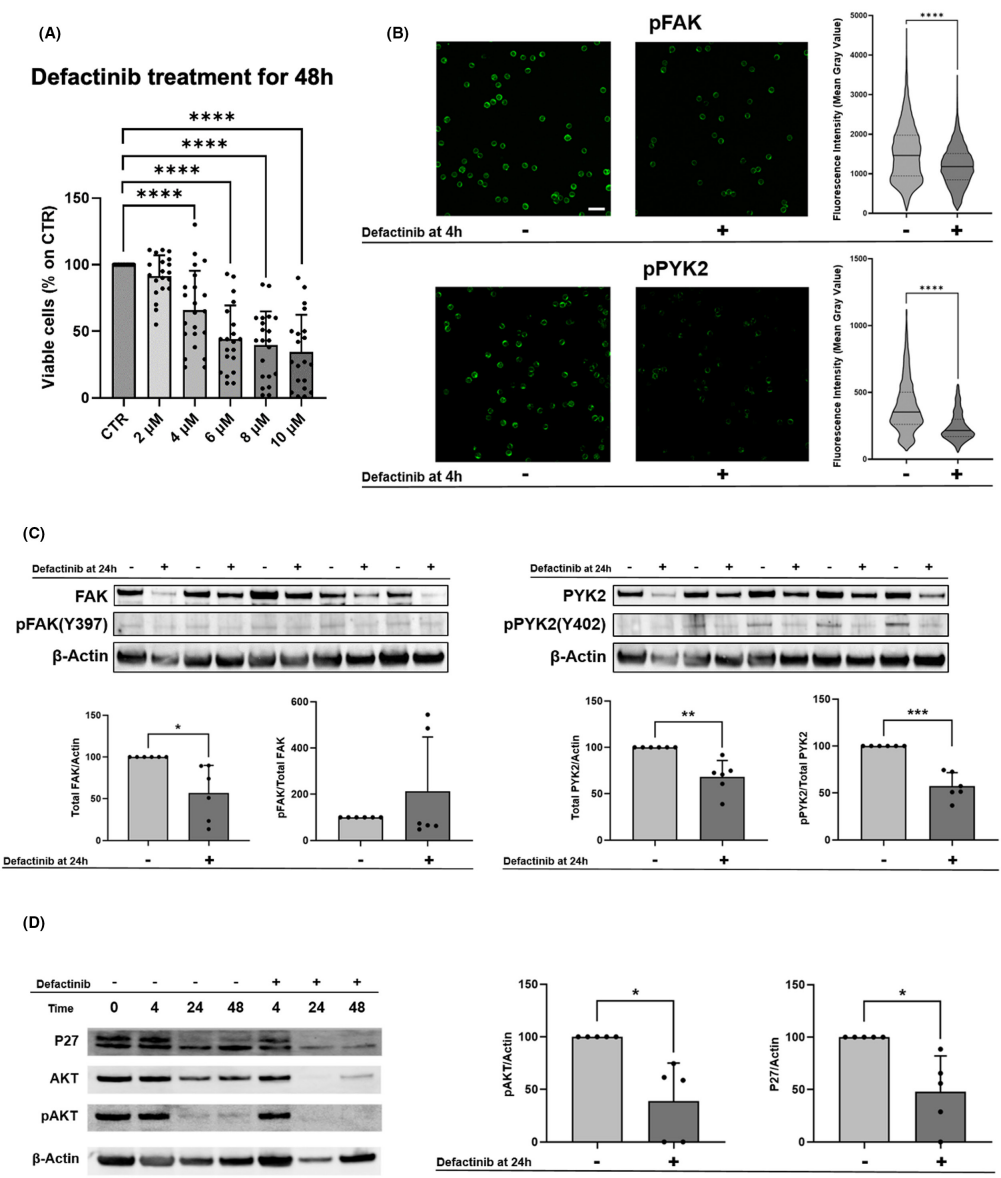
Defactinib treatment on primary CLL cells. (A) Viable cells were normalized on the controls. Defactinib has a dose‐dependent effect on cells after 48 h. In primary CLL B cells (*n* = 22), defactinib first significant toxic effect was found at 4 μM (*p* = 0.0016). (B) Left panel, FAK and pFAK IF analysis in patients' cells (*n* = 4). Representative confocal images of CLL cells stained for nucleus (Hoechst) and for either FAK or pFAK. Untreated and treated cells at 4 h were shown. Scalebar: 20 μm. Analysis of fluorescence intensity of pFAK, expressed in mean grey value, was normalized on total FAK intensity. Dots represent single values ± SD. **** *p* = <0.0001. Right panel, PYK2 and pPYK2 IF analysis in patients' cells (*n* = 4). Representative confocal images of CLL cells stained for nucleus (Hoechst) and for either PYK2 or pPYK2. Untreated and treated cells at 4 h were shown. Scalebar: 20 μm. Analysis of fluorescence intensity of pPYK2, expressed in mean grey value, was normalized on total PYK2 intensity. Dots represent single values ± SD. **** *p* = <0.0001. (C) Western blot on 5 CLL cases was carried out to assess protein's level FAK, pFAK, PYK2, pPYK2 in absence and in presence of defactinib at 24 h. Statistics shown are paired t test with the value of *p* = 0.024 for FAK, *p* = 0.0068 for PYK2 and *p* = 0.0007 for p‐PYK2. (D) Western blot was carried out to assess protein's level of P27 and pAKT. A representative western blot of P27, AKT, pAKT and Actin was shown, in absence and in presence of defactinib at 24 h (CLL cases analysed *n* = 5). In graphs, levels of proteins were normalized on untreated samples and also for total AKT for the pAKT measurement (*p* = 0.0193 for pAKT, *p* = 0.0270 for p27)

Firstly, we performed a viability assay testing different doses of the drug, ranging from 2 to 10 μM, on primary PB‐derived CLL cells (*n* = 22). Defactinib showed a dose‐dependent effect on CLL cells with a significant effect already at 4 μM (Figure 3A, *p* = 0.0016). We observed a high variability of response between patients though this did not correlate with known clinical or biological prognostic factors (based on Table 1 information) nor with FAK or PYK2 expression. Next, we aimed at assessing the direct effect of the inhibitor on both kinases. We performed both Western blot and immunofluorescence quantification to investigate pFAK (Y397) and p‐PYK2 (Y402) activation with and without the addition of defactinib at the concentration of 4 μM.

By immunofluorescence we detected a significant downregulation of the activation of both kinases after 4 h of treatment in all 4 patients analysed suggesting a specific effect of the drug (Figure 3B, *p* < 0.0001 for both). By western blot we observed for all patients analysed (*n* = 6) after 24 h treatment a clear downregulation of both total FAK and PYK2 protein level as well of p‐PYK2 while for pFAK we observed a high variability (Figure 3C, *p* = 0.024 for FAK, *p* = 0.0068 for PYK2, *p* = 0.0007 for p‐PYK2). We then investigated if defactinib treatment could lead to changes in downstream signalling survival pathways. Thus, we analysed pAKT activation and p27 (upstream regulator Cyclin‐D)^18^ expression in CLL cells obtained from 5 patients, before and after in vitro drugs exposure. We observed a significant pAKT and p27 decrease after 24 h upon treatment with defactinib (Figure 3D, *p* = 0.0193 for pAKT, *p* = 0.0270 for p27) with a high intra patients’ variability. This confirms the direct effect of the inhibitor on CLL cells survival. We then hypothesized that the microenvironment could exert a protective effect as observed for other cancers.^5^ To evaluate the effect of defactinib in the presence of leukaemia‐stroma interactions, we exploited the traditional 2D culture, thus seeding CLL cells in the presence of HS5 cells (4 cases), treating with 4 μM defactinib and collecting them after 48 h for flow cytometry analysis, where we used the Annexin V‐PI Kit to evaluate cell death and we could not detect any significant protective effect at this concentration and time point (Figure S1A). To test the ability of defactinib to mobilize CLL cells we took advantage of our established 3D in vitro model with a RCCS™ bioreactor,^25^, ^32^ which allowed us to study the mobilization and retention effects of defactinib in a scaffold in dynamic conditions ensured by the rotating vessels. The Spongostan™ scaffolds, made of gelatine, have a structure similar to the trabecular bone in the BM and they were populated with HS5 and CLL cells (*n* = 4). We then treated the co‐culture with Defactinib 4 μM for 4 h, and counted the cells outside and inside the scaffolds, to evaluate the mobilizing effects (Figure S1B). We did not observe a significant mobilization effect in contrast to the known impact on the mobilization of CLL cells from the tissue shown by other inhibitors such as BTK inhibitors.^25^

### 3.4 Defactinib treatment in a transplanted Eμ‐TCL1 mice model shows a reduction of CLL cells in the lymphoid tissues

**FIGURE 4:**
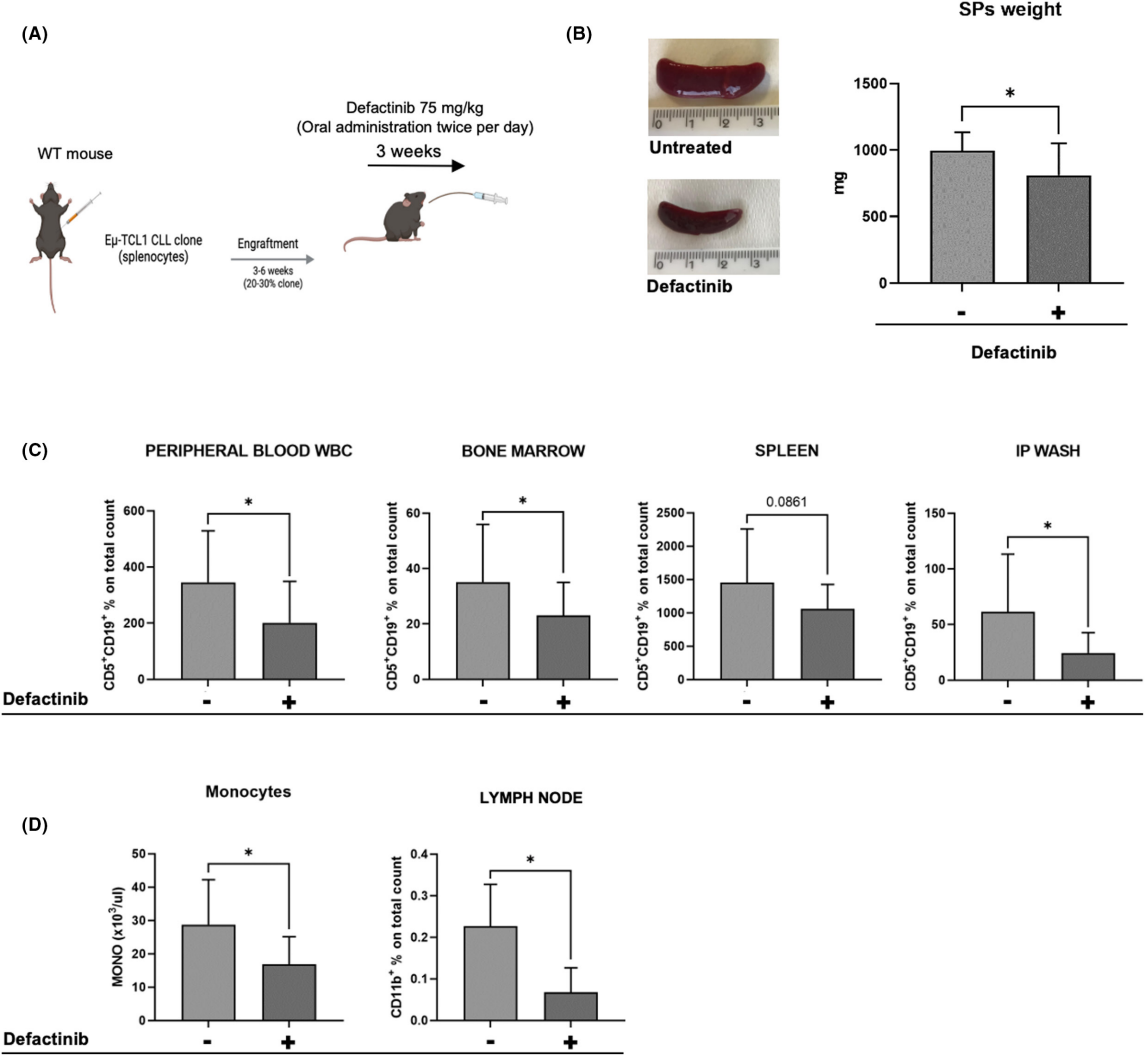
Defactinib treatment shows effectiveness in a CLL mouse model in vivo. (A) Experimental scheme of in vivo treatment created with Biorender (www.biorender.com). (B) Representative pictures of mice SPs. Quantification of weight and dimension (*p* = 0.0145). (C) Flow cytometry analysis of cells recovered from PB, BM, SP, IP‐wash to assess the percentage of malignant cells (CD5 + CD19+) in each department: the frequency on clonal leukaemic cells was significantly lower in the defactinib group (*n* = 18) compared to controls (*n* = 15) in all analysed tissues (BM, *p* = 0.049; PB, *p* = 0.0243; IP *p* = 0.0126) except for spleen (*p* = 0.0861). (D) Analysis of the macrophage counts of the two groups by CBC on K/μL at the end of the treatment: significant decrease in the number of macrophages (on cubic millilitre [K/μL] of blood) in the treated group (*p* = 0.0013). Flow cytometry analysis of CD11b + cells recovered from LN, showing a significant decrease (*p* = 0.0274) of the CD11b population (macrophages)

To investigate in vivo the role of FAK and PYK2 and the effects of defactinib in CLL, we utilized the transplantable CLL mouse model Eμ‐TCL1.^28^ Diseased mice were randomized in two groups to receive defactinib (*n* = 18) or vehicle as control group (*n* = 15) as depicted in Figure 4A. At sacrifice after 3 weeks, spleens from treated mice were smaller and lighter (*p* = 0.0145) compared to those from control mice (Figure 4B). Analysis of cells recovered from PB, BM, SP and intra peritoneal wash (IP) of both groups revealed that the frequency of clonal leukaemic cells (CD5^+^CD19^+^) as analysed by flow cytometry was significantly lower in the defactinib group compared to controls in all examined tissues (BM, *p* = 0.049; PB, *p* = 0.0243; IP *p* = 0.0126) except for the spleen (*p* = 0.0861; Figure 4C).

Finally, we were interested in evaluating the macrophage population in mice treated with defactinib compared to the controls, as it is known that macrophages support CLL progression^8^ and PYK2 affects both the morphology and the migration capacity of macrophages.^33^ Based on a Complete Blood Count (CBC) on PB samples, we observed a significant decrease in the number of macrophages (on cubic millilitre [K/μL] of blood) in the treated group (*p* = 0.0013) and a significant decrease (*p* = 0.0274) in the CD11b population (macrophages) in the lymph nodes as well (Figure 4D), confirming the importance of this putative target in the microenvironment for defactinib treatment.

## 4 DISCUSSION

The role of the microenvironment in CLL is of particular importance for both the pathogenesis of the disease and the emergence of drug resistance, also against novel more effective targeted therapies. Nowadays, it is clear that the study of the TME needs to focus not only on the biological interactions between tumour cells and their neighbouring cell components, but also on physical stimuli, which may trigger the activation of important signalling pathways for the disease course. With this intent, we investigated the role of focal adhesion kinases, FAK and PYK2, in the contest of CLL where their role and expression remain poorly investigated.

Firstly, we characterized FAK and PYK2 expression at mRNA level, revealing that PYK2 is expressed at higher level in CLL cells if compared to healthy B cells isolated from the peripheral blood. It is known that leukaemic B cells traffic between PB and primary or secondary lymphoid tissues where they find a supportive microenvironment sustaining their survival and activation^34^, ^35^ for this reason we analysed and compared both kinases expression also in lymphoid tissues. Interestingly we observed that both FAK and PYK2 are upregulated in healthy lymphoid tissues with respect to the PB while in CLL (tissues vs. PB) the level of expression is the same for both kinases, suggesting that CLL cells probably lose the capacity of healthy cells to regulate their expression during trafficking and homing. This is of particular interest considering that little is known about the CLL cells mechanism of retention and release from the protective niche present in the lymphoid tissues.

Along these lines, we found a significant correlation between FAK downregulation and progressive CLL cases and we observed that in the pre‐leukaemic MBL cases its expression is close to B cells isolated from healthy donors. While for PYK2 we showed that in MBL cases its expression is significantly higher with respect to healthy B cells confirming that PYK2 upregulation coupled with FAK downregulation could have a role in the transition from a pre‐leukaemic/stable disease to a more aggressive one and suggesting that a dysregulated balance of expression of the two kinases can occur during disease progression.

Knowing that both kinases play a major role in migration, adhesion and interactive structure formation such as podosomes, we investigated their expression, activation and localization. We found that all the cases analysed by western blot and immunofluorescence showed a constitutive activation of both kinases (although at variable level) and different intracellular localization. In particular, we observed pFAK present also in the nuclei of some CLL cells while p‐PYK2 was more punctuated in podosome‐like structures. In the future, studies are warranted to thoroughly address this different behaviour at the functional level in cells of haematopoietic origin and in other lymphoid malignancies where their role is still not clearly defined. Following these observations, we hypothesize that both molecules could represent a potential target for CLL cells in the microenvironment. We used defactinib, a selective inhibitor of FAK and PYK2 already used in clinical investigations for solid tumours and we exploited it in vitro and in vivo models, demonstrating an effect in all tested settings on CLL cells. Although we found a direct effect on survival associated pathways in all patients analysed, we also observed a high variability of response between patients that was not associated to the different level of expression and activation of the kinases, suggesting a more complex regulation. We also hypothesized that the inhibitor could have a mobilization effect on CLL cells impacting on their adhesion capacity to microenvironmental cells but we could not detect a clear effect in our 3D model that e previously demonstrated to be able to measure this effect for instance when cells are exposed to ibrutinib. Lastly, we exploited an in vivo CLL mouse model, where we confirmed defactinib pro‐apoptotic effect on CLL cells and interestingly we found that the macrophage population is a potential new target for the drug in this leukaemia, also knowing their crucial role in supporting CLL cells viability and protection from apoptosis. Collectively, these results helped in investigating for the first time the expression of both FAK and PYK2 in CLL, underscoring the need of further investigation of the TME, which supports leukaemia progression, and deeper understanding of each molecular player in the supporting niche, applying advanced in vitro models to better resemble real conditions. All of this, aiming at developing personalized and efficient therapeutic approaches for CLL patient care considering the possibility to combine focal adhesion inhibitors to currently used target therapies and/or to target other components of the supportive microenvironment such as macrophages.

## AUTHOR CONTRIBUTIONS

**Francesca Vittoria Sbrana:** Conceptualization (lead); formal analysis (lead); methodology (lead); writing – original draft (lead). **Benedetta Fiordi:** Data curation (supporting); methodology (supporting); writing – original draft (supporting). **Jessica Bordini:** Formal analysis (supporting); methodology (supporting); supervision (supporting). **Daniela Belloni:** Methodology (supporting). **Federica Barbaglio:** Methodology (lead). **Luca Russo:** Formal analysis (supporting); methodology (supporting). **Lydia Scarfo:** Investigation (supporting). **Paolo Ghia:** Conceptualization (supporting); writing – review and editing (supporting). **Cristina Scielzo:** Conceptualization (lead); data curation (lead); formal analysis (lead); funding acquisition (lead); writing – original draft (lead); writing – review and editing (lead).

## CONFLICT OF INTEREST STATEMENT

Authors declare no conflict of interest.

## Supporting information


Figure S1.

## Data Availability

The data that support the findings of this study are available from the corresponding author upon reasonable request.
