# Supplementary figures and images for: PYK2 is overexpressed in chronic lymphocytic leukaemia: A potential new therapeutic target
Source: J Cell Mol Med. 2023 Feb 6;27(4):576–86. doi: 10.1111/jcmm.17688 (PMC9930416; doi:10.1111/jcmm.17688)

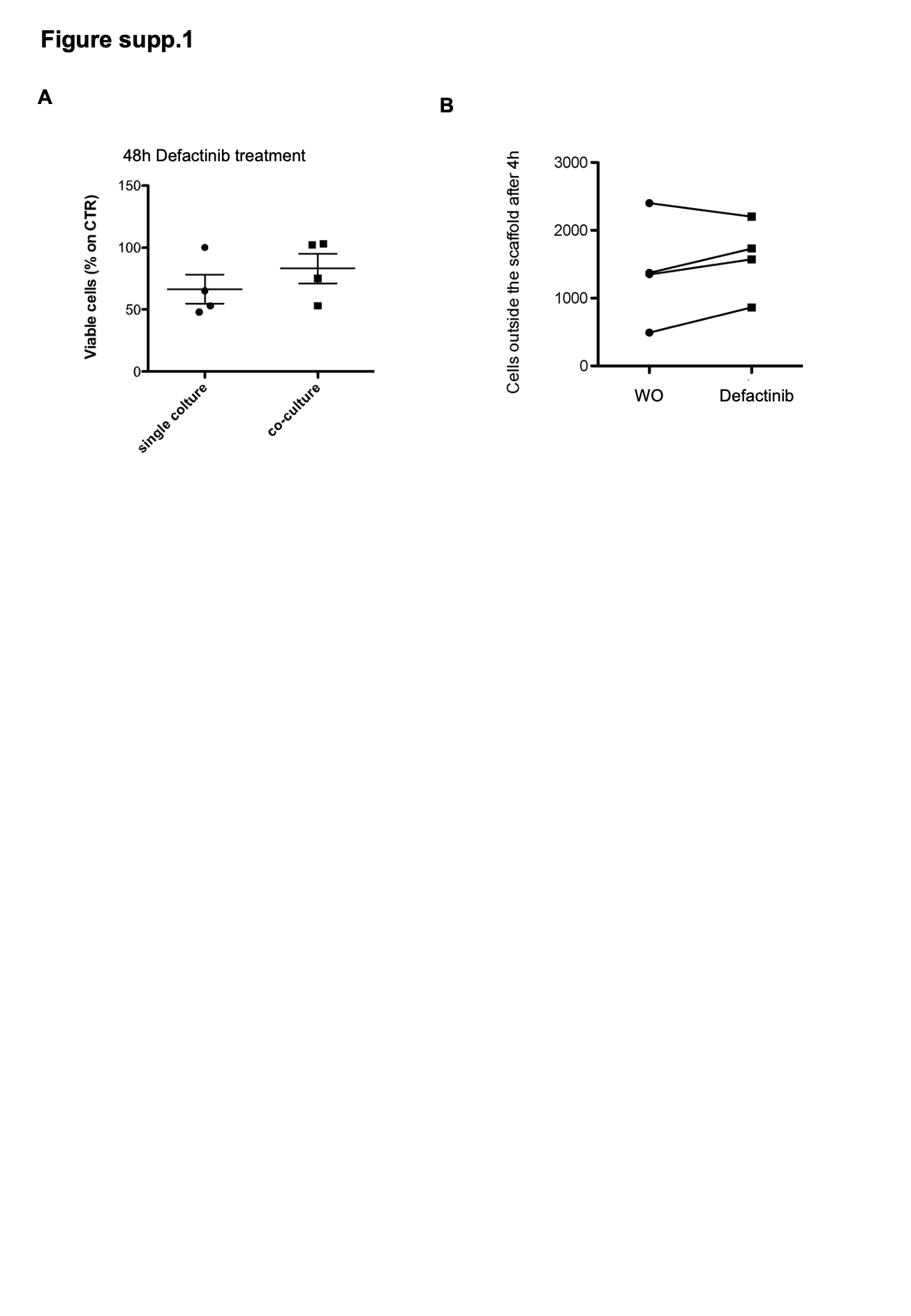

Supplement: Supplementary file 1 — Figure S1. [file JCMM-27-576-s001.tiff]
